# Social prescribing for suicide prevention: a proposed model for Australia

**DOI:** 10.3389/fpubh.2025.1547468

**Published:** 2025-03-24

**Authors:** Sarah Dash, Stella McNamara, Maximilian de Courten, Rosemary Calder

**Affiliations:** Australian Health Policy Collaboration, Victoria University, Melbourne, VIC, Australia

**Keywords:** social prescribing, suicide prevention, link worker, service model, community, primary healtcare

## Abstract

Suicidality is a devastating and burdensome experience that can be a result of complex psychological, biological and social factors. Social prescribing could be well suited to address the diverse non-clinical needs of people experiencing suicidality. International and Australian evidence indicates social prescribing provides an effective and acceptable approach. To address suicide risk and rates in the Australian community, community-based approaches that are visible, readily accessible and that address complex social, practical or non-medical needs are needed. We propose a social prescribing model for suicide prevention that could be implemented in Australia either as a specific purpose service or within existing social prescribing trials, with relevant modifications tailored to suicide prevention. Drawing upon evidence from the literature and a panel of social prescribing experts, we make practical recommendations for implementing a social prescribing model for suicide prevention in Australia, and discuss some of the system-wide requirements to support access and scaling up of these models.

## 1 Introduction

Social prescribing is an approach, at the primary health care or community level, that identifies individual need and connects them with non-medical, community or social support to address social, emotional or practical needs to improve overall wellbeing and health (1). Social prescribing originated in the UK in the 1980s and though it initially aimed to target social isolation in aging populations, it has expanded internationally and to target a range of populations. Co-creating a social prescription (2) between the link worker—an employee who liaises with the client, community organisations and where relevant, health professionals—and the individual is a central component. Social prescribing models commonly involve general practioners (GPs) (also referred to as primary care physicians) who refer individuals to a social prescribing link worker or link worker service who works with individuals to co-create a social prescription plan and connect them to local, non-clinical services appropriate to their needs. Models of social prescribing have been developed and trialed internationally (3) and more recently, in Australia (4–6). Emerging literature supports the benefits of social prescribing for health and wellbeing (7–11), as well as general acceptability of social prescribing for both clients (7) and practitioners (12).

Social prescribing has been found to have benefits for mental health by improving social, psychological and emotional wellbeing (13). Social prescribing can also play a role in suicide prevention by providing individuals with access to community-based support services that can help address the underlying social determinants of health that contribute to suicide risk (14). Social prescribing can help address social isolation and loneliness, which are known risk factors for suicide (15). Emerging evidence for social prescribing trials in Australia have demonstrated preliminary effectiveness and acceptability (16).

Our recent rapid review summarises key considerations for social prescribing for suicide prevention (16). Briefly, these include additional monitoring and support of referrals, including warm (or assisted) referrals, among those at suicide risk (17–20). Additionally, models should include support for social prescribing at each level of health and social care service provision, the individual level, referral pathways and health/social infrastructure (21). Lastly, successful models require and additional training and resourcing of link workers to support suicide prevention (22). Importantly, social prescribing models should be co-designed with communities to ensure that community needs are met, to empower communities and to support stakeholder buy in Thomas et al. (23).

Our aim in the current project was to identify the potential for an evidence and expert informed model of social prescribing for suicide prevention in Australia and to provide guidance on the components and structure to support implementation of a social prescribing for suicide prevention model within existing health and social infrastructure (24).

## 2 Context

Our proposed model incorporates evidence from academic and grey literature and an expert advisory panel. The panel consisted of a general practitioner, an employee of an existing social prescribing trial and a lived experience adovocate, and consultation included first via one-on-one interviews to scope the work, followed by an online panel meeting to validate the model with individual expert review and feedback to review and finalise the model. This model is intended for implementation in Australia among existing health and social support systems.

## 3 The model: a social prescribing model for suicide prevention

### 3.1 An overview of the proposed model

There are three foundational aspects of this model. Firstly, there must be means of identifying, supporting and referring people who are at risk of suicide and a relevant chain of community touchpoints to support this. Secondly, the definition of community where this model is implemented needs to be carefully considered, and support must be layered (e.g., extending beyond the boundaries of a defined community) where need requires. Finally, link workers and a warm (assisted) referral are critical to the success of a social prescribing model for suicide prevention. A link worker must be a central, well-known resource that is appropriate for the characteristics and needs of the community (such as culturally relevant) and that community actors (e.g., employers, clinicans, family members, youth centres) can connect individuals to.

**Figure 1:**
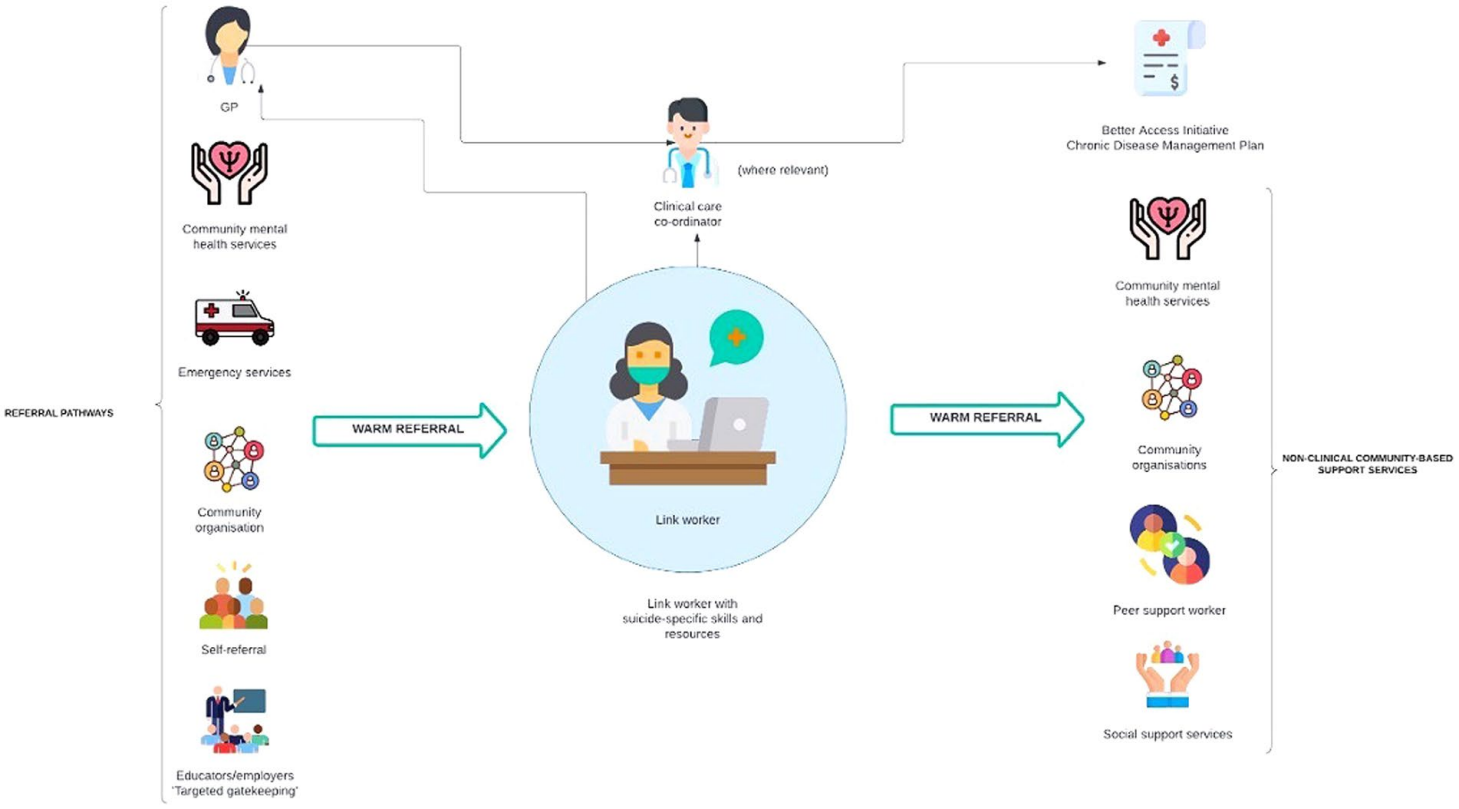
Proposed model of social prescribing for suicide prevention in Australia.

These elements and other key considerations for implementation are illustrated in Figure 1 and described below.

### 3.2 Host location

The proposed model is intended to complement existing health and social support infrastructure, while incorporating important modifications required to support scale-up. For example, for a suicide prevention social prescribing service model that is immediately accessible to all in the relevant community, a link worker would be best located in a well-established community hub. In smaller, possibly isolated communities, this may be a GP’s practice, or it may be a particularly appropriate community organisation; whereas in larger catchments, a network of relevant locations throughout the community, within one or more community organisations, may be appropriate. In all communities, the location of link workers should be determined through a careful co-design approach with key community stakeholders. Importantly, social prescribing models for suicide prevention must have a level of flexibility, allowing for adaptability in sourcing support beyond defined catchment areas when necessary (e.g., LGBTQIA+, First Nations peoples may require support and a sense of ‘community’ from outside of defined geographical community boundaries).

Primary Health Networks (PHNs) are independent organisations with geographically defined boundaries in Australia that are funded by the Australian government to assess the needs of their communities and to commission and support relevant primary health services for them (25). Given that suicide prevention is a national health priority and evidence that communities are significant in suicide prevention initiatives (26, 27), PHNs are a logical platform for and could serve as commissioners of social prescribing for suicide prevention. One or more partner organisations (e.g., community organisations, Local Government Authorities (LGAs) or GP practices) would be commissioned to provide the service. Commissioning requirements would include performance measures and evaluation with accountability to a community governance structure comprising stakeholder community organisations, the commissioning organisation and relevant service providers in the catchment.

The expert panel members largely agreed that GPs are a logical location for social prescribing. GPs are familiar, knowledgeable and many already practice various forms of social prescribing. Evidence indicates that there is increased contact with primary care prior to suicide (28, 29), suggesting that GPs are well placed to intervene in suicide risk. However, a suicide prevention model also has the challenge of reaching people who may be less likely to be engaged with a GP or clinical services than those with physical health concerns. Additional mechanisms in a social prescribing model for suicide prevention (e.g., self-referral, community member supported referral) are required to support those at risk of suicide. Importantly, co-creation with community can help to determine referral pathways and the link worker location that best meet the needs of the community.

### 3.3 Link worker as the central piece

Evidence from social prescribing literature and the advice of the project expert panel both support the role of the link worker as a central tenet of a social prescribing for suicide prevention model. The success of social prescribing relies on the availability and accessibility of a link worker to provide connections between clinical and community care that are not readily made and that may act as barriers for many individuals.

The link worker(s) are a central referral point for GPs, community organisations, community mental health services, first responders, key stakeholders working with high-risk groups (e.g., employers, teachers) and those who wish to self-refer. Though a link worker is a feature of broader social prescribing models, a link worker working within suicide prevention should have specific suicide-related training and understanding of relevant social supports within the community. A link worker must have specific knowledge of the characteristics and needs of the community, a comprehensive understanding of the support services within that community (or beyond, as relevant) and strong, ongoing relationships with both referral and support services. In some cases (e.g., smaller communities), a link worker role may be taken on by an existing staff member (e.g., general practice nurse). In these situations, role delineation between the scope of the link worker role and any other responsibilities (e.g., managing clinical care) must be established. Additionally, a link worker must also be connected to supports for link workers directly, including connection to or embedding within a network (e.g., a GP practice or community organisation in which they are located), for both oversight of their role and support services, as needed.

Importantly, a link worker may also refer back to a GP for clinical care if required. This may mean that some individuals receive clinical care and/or access to clinical management schemes (e.g., Better Access Initiative that gives government rebates to help people access mental health professionals and care, or the Chronic Disease Management Plans that are to help eligible health professionals coordinate health care for patients with chronic medical conditions) in combination with non-clinical support.

### 3.4 Peer worker as potential support

Inclusion of a peer worker within the link worker service, should be considered. Peer workers are recognised as important supports in both mental health and suicide prevention. The Australian Government Department of Health and Aged Care guidance on the role of the peer workforce in mental health and suicide prevention states that PHNs can support better outcomes by promoting and supporting the employment of peer workers as part of multi-disciplinary teams providing person centred support and recovery-oriented and trauma informed care. The peer workforce includes both consumer and carer peers (30). Peer workers have been found to be effective in supporting access to physical health care for people with mental illness and a study of consumers and carers views of peer worker support found that individual peer worker roles were considered to have significant potential value in facilitating access to health information and in assisting with motivation, amongst other benefits (31).

### 3.5 Warm referrals

Warm referrals – as opposed to signposting, in which a person is provided with advice and expected to follow that advice without assistance—are a more personalised and involved approach, including the handoff of individuals between members of a care team that takes place in front of and with the person (32). Warm referrals must go above encouragement, provision of information or sharing contact information for support services. This is in line with evidence that identifies that trust, rapport and additional support is required for people at risk of suicide or experiencing suicidal distress. Specifically, evidence suggests that there are relationships between social capital and social trust and rates of suicide (18, 19). Additionally, higher levels of social isolation are also associated with elevated suicide rates (33). This evidence highlights the additional social and relational barriers that may exist for people at risk of suicide which must be addressed with additional supports. The absence or compromise of warm referral is likely to be a significant barrier to the success of a suicide prevention social prescribing model.

## 4 Implementation

### 4.1 Funding

It is important to note that PHNs currently allocate funds to support preventive health initiatives, and a social prescribing for suicide prevention model would fit well within this capability. PHN organisations could engage a partner/provider to deliver the model. However, PHNs require additional and specific funding to adequately implement an SP service.

Importantly, link workers must be appropriately funded for their salary and infrastructure support and to cover the cost of establishing and maintaining a wide referral network and to provide followup support for participating individuals. An essential component of funding support to be considered is ongoing link worker support to clients as determined by individual need and a budget to meet the cost of access by individual clients to community supports that require a financial membership, sessional fee or subscription.

In addition to supporting individuals to access community services where they may incur a gap fee, funding approaches must also consider the impact to community services who will receive increasing numbers of referrals. Having adequate resourcing (e.g., staff, space) for these community services to manage referrals from social prescribing is integral to the model’s success. Although this funding sits outside the social prescribing model itself, community services that will form social prescribing referral networks must be appropriately funded to support the success and sustainability of the model. Development of a funding framework for social prescribing needs to be co-designed with key stakeholders. Development of a funding framework for social prescribing should be informed by pilot projects with evaluation of the individual health outcomes and the social return on investment.

### 4.2 Referral pathways

Several inbound referral pathways to a link worker must be available for best coverage of those who need support. GPs offer a direct referral for patients who may visit a GP for a health or suicide-related reason. In addition, various other inbound referral pathways must be in place to offer comprehensive community coverage to those who need support. There should be ‘no wrong door’ for referrals. Community mental health services, community organisations, first responders and members of the community themselves must all be able to directly refer to a link worker. Additionally, to account for the likely disconnect between individuals at risk and engagement with services, individuals in high-risk environments (e.g., employment, education) should be viewed as ‘connectors’ who have knowledge of their local link worker and the pathway available for referring someone at risk. Importantly, this model includes self-referrals which may include individuals at risk of suicide connecting directly with a link worker or being connected via friends and family. Given the urgency of some suicide prevention, self-referral to a link worker does not replace emergency services or urgent support [e.g., Lifeline (34)]. Self-referral to a link worker does also not preclude access to clinical services where relevant, and a link worker may also connect individuals with clinical health or mental health care. This emphasises the importance of appropriate skills and training for the link worker role. Lastly, referral pathways may differ between communities and should be co-designed based on the needs and infrastructure of each community.

### 4.3 Evaluation and governance

Evaluation of trials of social prescribing for suicide prevention must consider the complex nature of suicide risk factors and be cognisant that the benefit of social prescribing and community supports for individuals at risk of suicidal behavior are unlikely to be evident in the short term. Testing of this model should be designed intentionally to develop an evaluation framework for social prescribing in suicide prevention that considers the:

- Implementation process,
- Effectiveness of engagement of key stakeholders and community supports,
- Appropriateness and effectiveness of referral pathways within the community, and develops relevant measures of individual outcomes or benefit that are applicable to a time limited trial project.

Lessons from the National Suicide Prevention Trial – Final evaluation report (35) should be taken into the design of an evaluation framework. These findings, including considering the needs of at risk groups, engaging the right stakeholders, and the importance of whole-of-government support should inform subsequent scale-up and and evaluation.

Importantly, performance indicators for the link worker role should identity the role scope for stand-alone link worker positions and for the role when it is undertaken by a person with an additional clinical or other role related. Having a clearly defined link worker role can help to provide guidance and boundaries for instances in which the link worker becomes involved in clinical management, to establish role delineation from a clinical case manager.

Overall, a comprehensive evaluation of a social prescription trial should include a combination of quantitative and qualitative methods to assess its effectiveness in improved health outcomes for individuals, as well as its cost-effectiveness. It is important to work with stakeholders, such as healthcare providers, community organisations and consumers, to ensure that evaluation is rigorous and relevant.

A social prescribing model for suicide prevention must be accountable to a governance structure through regular reporting. An appropriate governance structure would involve regular meetings with a community steering committee and regular collection of key metrics, including program uptake, case presentation, types of referrals, effectiveness of the link worker role scope relevant to community needs. This requires collaboration between healthcare providers, community organisations and other relevant community stakeholders to ensure that the social prescription program is effectively implemented and meets the needs of the target population. The World Health Organization toolkit outlines the steps required to introduce a social prescribing scheme (36). It can be used by implementing organisations, community healthcare and long-term care facilities, mental health and healthcare workers, among others.

### 4.4 Suicide prevention could be trialed within existing social prescribing programs

From a pragmatic, implementation perspective, a social prescribing model for suicide prevention should be embedded within an existing or broader social prescribing program with additional support such as a link worker with specific suicide-related training and additional mechanisms to engage at risk community members. These could be “added in” to existing social prescribing programs and evaluated against specific suicide-related risk factors and outcomes. The model should also be integrated with other existing suicide support, such as suicide aftercare programs [e.g., Next Steps (37)]. Additionally, a social prescribing model for suicide prevention could be developed independently to existing trials or programs to meet any unmet community need. This may mean that a community organisation identifies a need and creates a backbone structure (e.g., a ‘home’ for this model and/or a link worker), which supports community ownership of the implementation.

## 5 Discussion

This case study presents a model of social prescribing for suicide prevention that builds on existing evidence, expert opinion, and is tailored to the specific needs of suicide prevention. The key components of this model include multiple referral pathways, including self referral, the importance of a warm referral and a link worker as a central pieces. As discussed, the ‘host’ and boundaries of this model may be unique to each community, though we make pragmatic recommendations based on utilizing existing infrastructure, such as Primary Health Networks. Essentially, the implementation must be co-created with communities to ensure that the needs and preferences of the community are central. Additionally, the resourcing of link workers and the pathways and services connected to them (e.g., community services who may experience an increase in number of users) must be carefully considered if this model is to be sustainable. This models serves as a framework to be adapted to meet specific needs of different communities around Australia.

## 6 Conclusion

Our proposed model makes practical, evidence and expert informed recommendations for establishing social prescribing models for suicide prevention in Australia. The movement towards providing non-clinical prevention and treatment for complex health and mental health concerns is in line with a holistic, well-connected care. Given the complexity of suicidality and the evidence for social prescribing in Australia, social prescribing for suicide prevention seems a logical and pragmatic model.

## Data Availability

The original contributions presented in the study are included in the article/supplementary material, further inquiries can be directed to the corresponding author.
